# Supplementary figures and images for: Comparing audio- and video-delivered instructions in dispatcher-assisted cardiopulmonary resuscitation with drone-delivered automatic external defibrillator: a mixed methods simulation study
Source: PeerJ. 2021 Jul 15;9:e11761. doi: 10.7717/peerj.11761 (PMC8286704; doi:10.7717/peerj.11761)

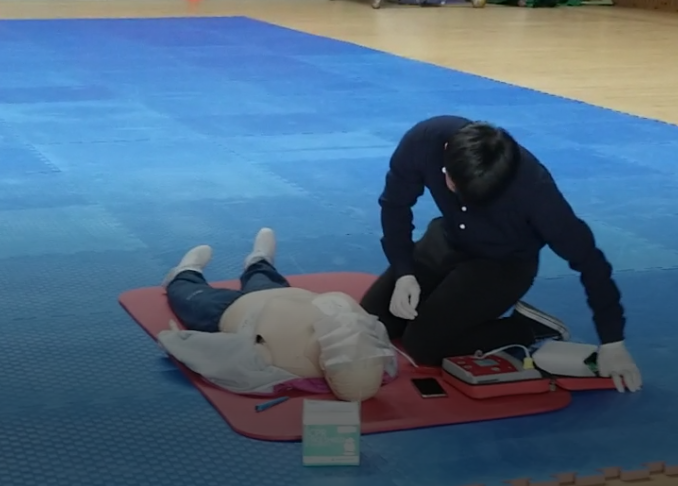

Supplement: Supplemental Information 2 [file peerj-09-11761-s002.png]

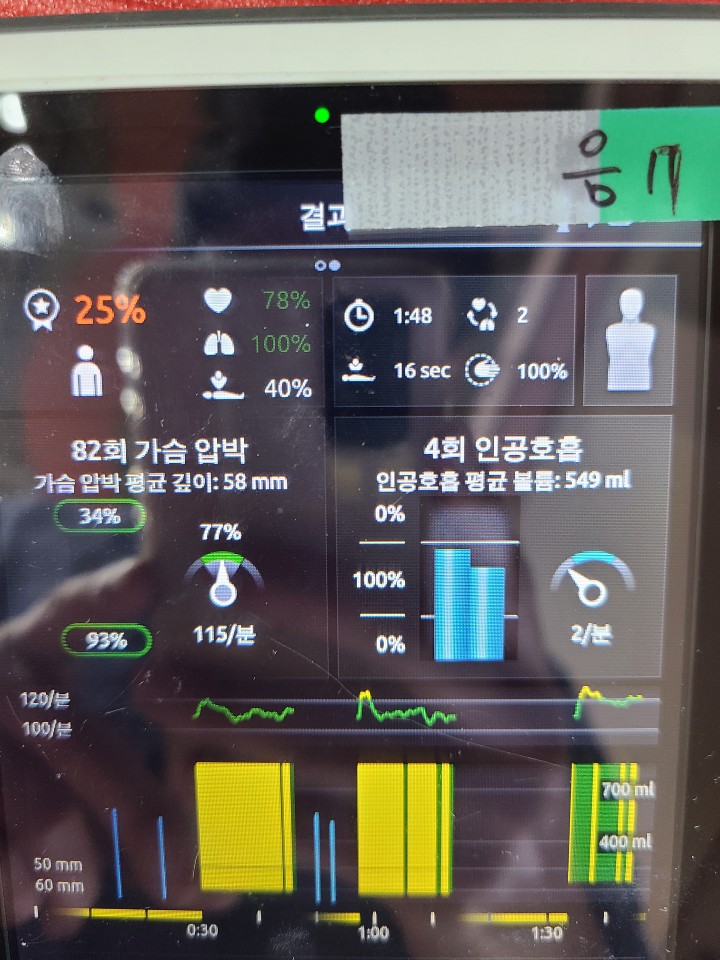

Supplement: Supplemental Information 3 — Sim Pad Skill Reporter [file peerj-09-11761-s003.jpg]

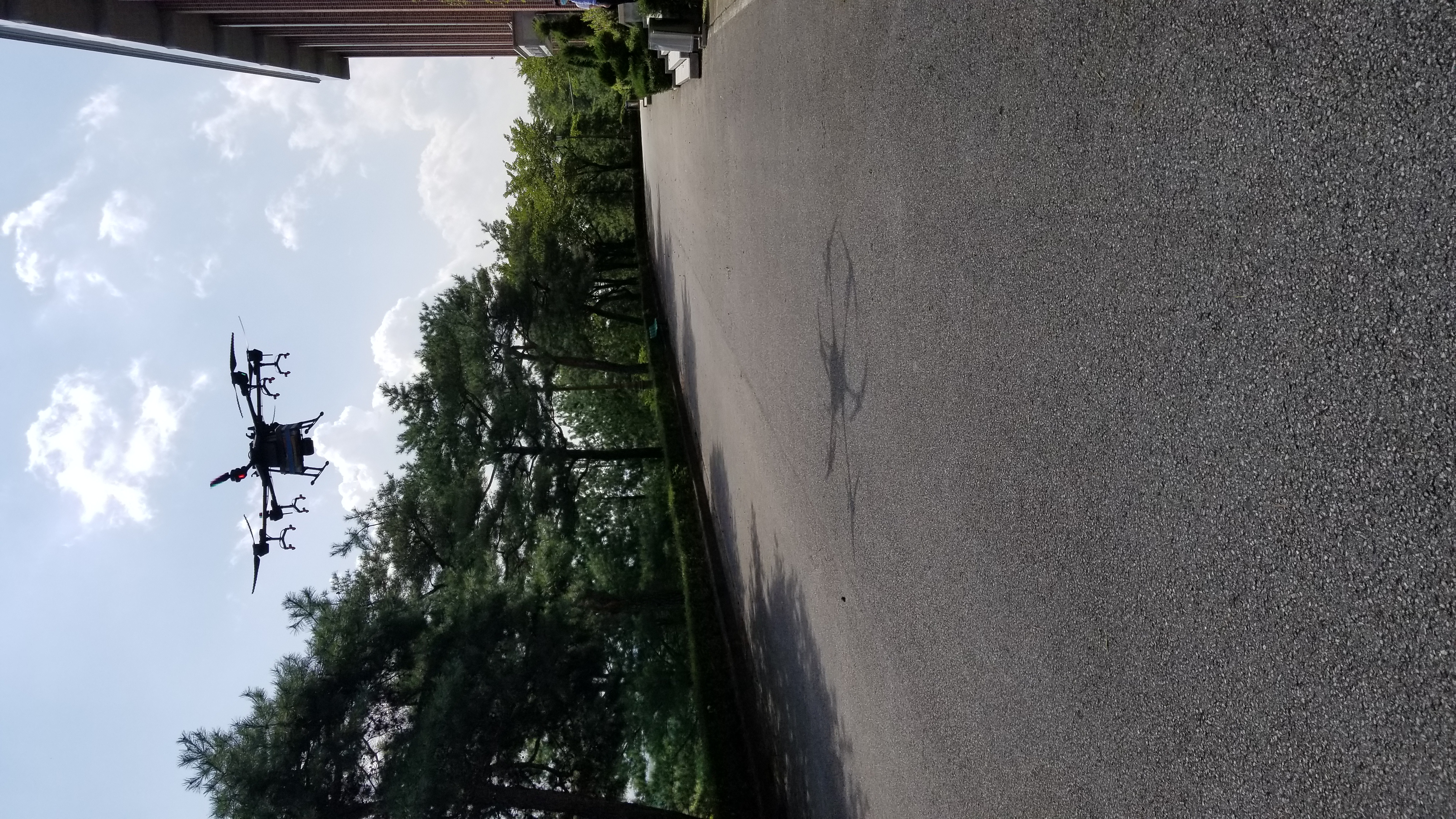

Supplement: Supplemental Information 4 [file peerj-09-11761-s004.jpg]

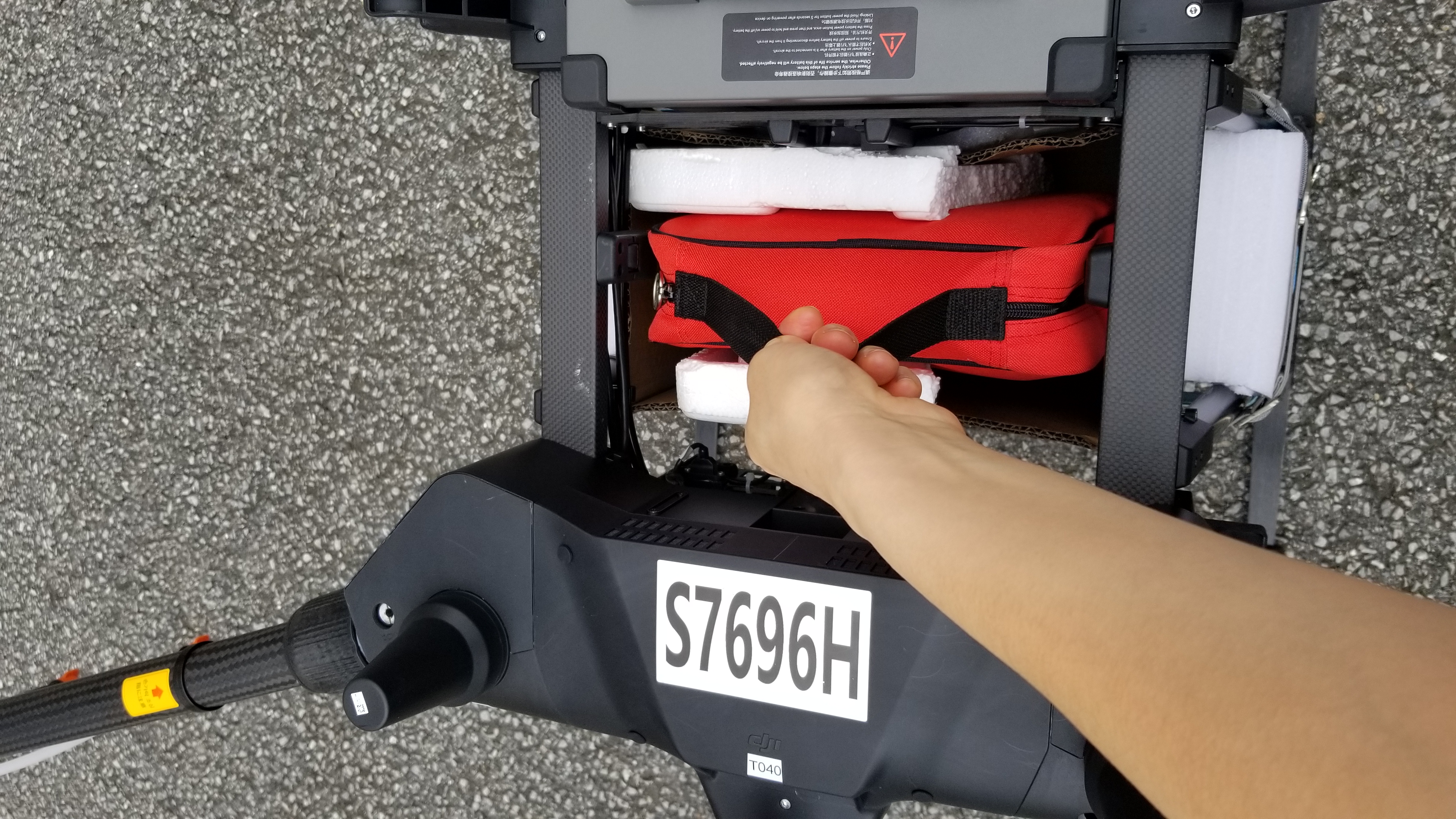

Supplement: Supplemental Information 5 [file peerj-09-11761-s005.jpg]
